# ‘I cannot say that he is of unsound mind’: the case of Lunatic Richard Lea

**DOI:** 10.1017/mdh.2026.10057

**Published:** 2026-04

**Authors:** Mishka Wazar

**Affiliations:** History, Princeton University, USA

**Keywords:** Cape Colony, madness, psychiatric history, South Africa, lunatic asylums, South African War

## Abstract

This paper examines letters from the casebooks of the Valkenberg Lunatic Asylum in the Cape Colony during the South African War. Valkenberg was opened in 1891 in Cape Town, and was the only asylum established exclusively for white patients in the Cape. The South African War took place between 1899 and 1902, and several soldiers serving in the War were treated at Valkenberg during this period. The letters were written by a male patient who used bureaucratic and legal channels to claim his sanity and secure release from the asylum, showcasing a rare example from the archive of a patient’s voice as well as a view into the inner workings of a colonial asylum in South Africa. These letters allow a view into the personal lives of patients and attendants, the medical rules doctors followed, and instances of racism, unexpected solidarity, and loneliness. Analysing these letters reveals the changes taking place in a turbulent South Africa, including the tensions and conflicts of a country at war, the racism and nationalism of early twentieth-century South Africa, and the violence present within the asylum network. By examining letters written directly by a patient, which give voice to a perspective that official institutional records would not ordinarily allow, this paper seeks to contribute to the literature on patient voices in the history of psychiatry.

At the turn of the twentieth century in the Cape Colony, a British Sergeant serving in the South African War wrote several letters. This case should not be remarkable. The South African War broke out on 11 October 1899, and hundreds of thousands of British men and women were involved in the conflict against the Boers and allied Black participants.^1^ Many of these people documented their experiences of the war through letters and diaries, and several scholars have analysed these texts for what they contribute to understanding the conflict and how it changed the face of South Africa.^2^ What is remarkable here is that this soldier was also a patient in the Cape’s network of lunatic asylums. This soldier-patient is named Richard Lea, who wrote from the Valkenberg and Robben Island Asylums in Cape Town during the colonial period of the early twentieth century.^3^

**Figure 1.**
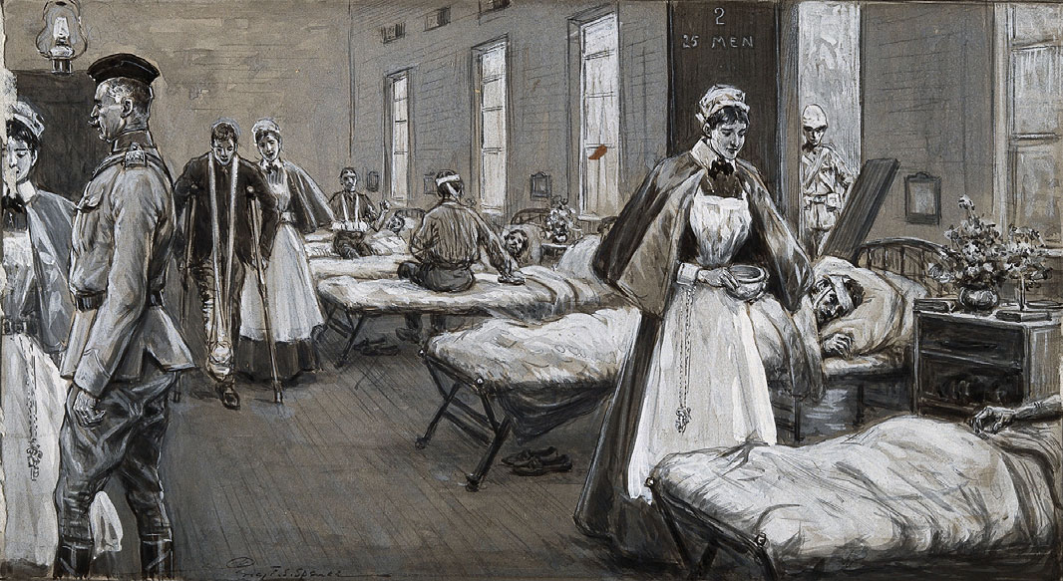
Boer War: a full ward in the Wynberg Military Hospital, South Africa, with nurses attending the wounded.^27^

**Figure 2.**
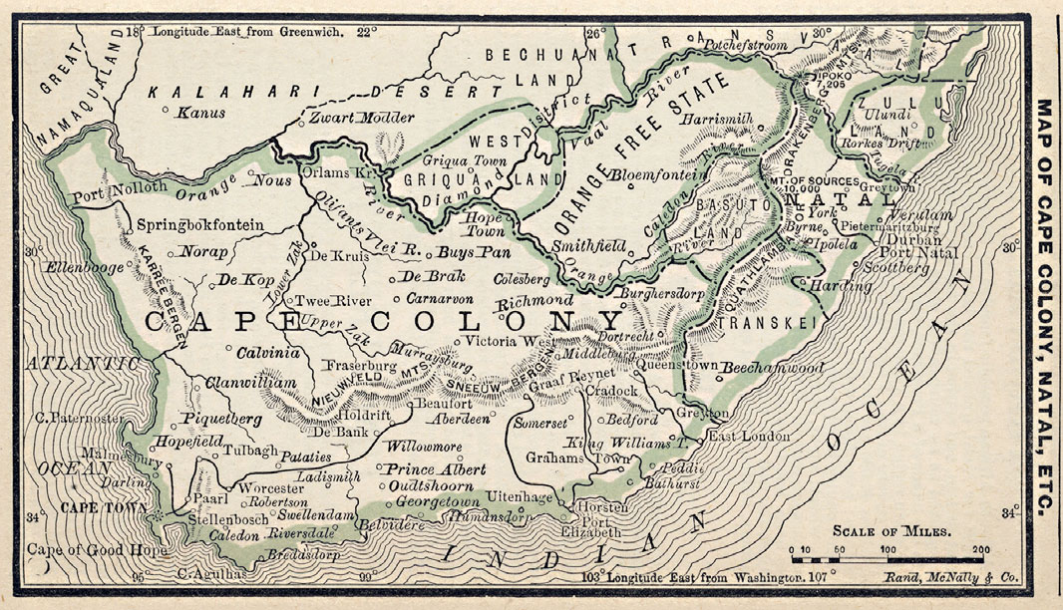
Map of Cape Colony, 1887.^57^

The case records of Richard Lea’s detainment are striking because, as many scholars have argued, it is notoriously challenging to find archival evidence of psychiatric patients’ voices.^4^ Medical professionals working at the turn of the century largely dismissed the voices of the insane as ‘nonsense’, failing to record or preserve their words in case notes, leading to a dearth of patient testimony in histories of psychiatry.^5^

However, since Roy Porter’s 1985 article ‘The Patient’s View: Doing Medical History from Below’ called for bringing the histories and voices of patients to light, historians of psychiatry have produced many patient-centred histories and sources.^6^ Louise Wannell has written about the practice of letter-writing among the friends and family of patients at the York Retreat asylum, showing their involvement in the life of the patients.^7^ Catharine Coleborne’s work explores how asylum correspondence provided a means for intimacy and connection between interlocutors across spatial distance.^8^ Effie Karageorgos and Rory du Plessis have worked on case records involving delusional content expressed by patients in asylums in Australia and South Africa.^9^

Still, scholars have generally relied on institutional records of patients, which primarily offer the perspective of doctors and other officials.^10^ Case notes are valuable to the historian of psychiatry, providing information about the demographics within asylums and the perceptions of gender, race, and class among doctors and broader society, as well as insight into how asylums were run.^11^ Asylum reports included tables describing the causes of insanity, which often differed between men and women.^12^ However, these sources do not entirely reveal what patients experienced in these institutions, and there are often issues of inconsistency, bias, and censorship in the narratives produced by doctors. The patient’s voice is mediated through the gaze of the doctor, offering only a distorted view of patient experiences.^13^

In this paper, examining letters written directly by a patient provides an opportunity to recount one individual’s experience of institutionalisation, giving voice to a perspective that official institutional records would not ordinarily allow.^14^ In the literature on South African psychiatry, several scholars have used ‘unexplored’ sources to produce histories. Sally Swartz, the eminent historian of psychiatry in South Africa, has argued that there are few archival sources available that document women’s experience of institutionalisation.^15^ Swartz made use of asylum statistical tables from annual reports to produce a history of women and gender in South African asylums.^16^ du Plessis has examined different sets of photographs from the casebooks of the Grahamstown Lunatic Asylum to argue that asylum officials used photography to project a positive public image of the asylum while undermining Black resistance to confinement.^17^ While the literature on this subject has focused on the Cape, Julie Parle has made a significant intervention in this literature by examining the few sources on the history of psychiatry in the Natal Colony.^18^ Despite these ground-breaking studies in the literature on South Africa, there remains an absence of accounts of patients’ experiences in asylums, as many patients did not speak English and could not write.^19^ This paper seeks to expand the history of psychiatry in South Africa by offering more representation of patients’ voices and lives.^20^

Richard Lea wrote twelve letters to asylum superintendents, colonial authorities, lawyers, and newspaper editors over nine months, insisting on his sanity and demanding his release from the Cape’s asylum network. His letters detail his living conditions in the asylum and his daily life and experiences of violence and solidarity. While not without their limitations, Lea’s letters give insight into the mobilities and trajectories of his life before and during his confinement and, more broadly, analyse the geographical, national, and racial tensions of the asylums and South Africa.^21^

## Valkenberg

In the third year of the South African War, Sergeant Richard Lea was admitted to Valkenberg Lunatic Asylum in the Cape Colony. Lea was a British sergeant serving in the regiment Kitchener’s Horse, and a few weeks before being admitted to Valkenberg, he fell off his horse in the Transvaal. Due to his injuries, his unit sent him to Wynberg Military Hospital in Cape Town.^22^

During the South African War, Cape Town was the centre of British military organisation, forming the site for many military hospitals.^23^ Wynberg Hospital, also known as No. 1 General Hospital, was built from vacated buildings in the Wynberg Military Camp and opened in 1899.^24^

The facilities and provisions at Wynberg were better than at many other hospitals, and the medical staff would have treated Lea well. However, something happened to Lea while he was recovering from his fall. According to a report in the *Cape Times* later that year, Lea’s ‘mind was temporarily affected’, and his doctor transferred him to Valkenberg on 16 January 1901.^25^ Legal documents at this time show that two doctors certified Lea as a lunatic for his transfer to Valkenberg. The first medical certificate, signed by Dr John D, states:

He is very strange in humour, at all times very excited. Is continually writing letters to people in authority. Walks up and down the ward talking to himself. Eats and sleeps well. Indian in his habits.^26^

Lea had been living in Bombay before joining the war, and his family remained there. Dr Davis’s remark that Lea was ‘Indian’ in how he practised hygiene points to a concern among colonial physicians at this time that European settlers were vulnerable to physical and mental illness through living in the colonies and interacting with indigenous populations.[Fn fn27] ^28^ It was believed that if the settler began to imitate the colonial subject – by ‘going native’ – they would experience physical and mental degradation.^29^ If Lea had begun to imitate Indian way of life, this was one sign that he was descending into madness.

The second medical certificate, signed by Dr David P, claims that Lea believed ‘that his comrades wanted to strangle him. His appearance is nervous with wild-looking eyes’. Other medical and admission documents state that Lea was considered dangerous to others, but his cause of insanity was listed as ‘unknown’.^30^

Karageorgos notes that many Australian men who enlisted in the South African War developed war-related trauma after their service, experiencing delusions about being followed by enemies and behaving violently in Australian asylums.^31^ Similarly, du Plessis shows that patients in the Grahamstown Lunatic Asylum experienced delusions related to the war.^32^ The South African War was a traumatising experience for many participants and civilians in the country, and changed the face of South Africa. Lea had been in the country for 12 months before falling off his horse and being sent to Wynberg.^33^ There are no sources available to show what he went through during his active service, but it does not seem far-fetched to imagine that he experienced threats and danger to his life. Did these experiences cause the behaviour that concerned his doctors enough to transfer Lea to Valkenberg?

Valkenberg Asylum opened in 1891 and was the only asylum established exclusively for white patients in the Cape.^34^ Here, Lea would have encountered an environment similar to asylums in Britain at the time. A little over a month after he was admitted to Valkenberg, Lea wrote a letter to the Colonial Secretary of the Cape asking to ‘make enquiries from this Asylum in giving me my liberty. I’m in good health and able to take care of myself’.^35^ It appears that he received no response, and a few weeks later, he wrote to the Colonial Secretary another letter, complaining about the conditions in the asylum. He described the bedrooms as ‘wretched’ and lamented the lack of ventilation. He claimed that Valkenberg’s Medical Superintendent, William J. Dodds, knew nothing about his patients and relied on his attendants for information. Lea’s primary complaint is with the attendants, who he says are ‘mostly Dutch and German *Pro* *Boers,* whose ambition is to persecute the British soldiers whose misfortune is to be sent here’. He continues, ‘I’m a born Englishman […] I served 12 years & 5 months in the Army. Is it right for Dr Dodds to detain me any longer here when I’m in good health? He is driving me to commit myself’.^36^

In Britain, medical staff were allowed to open patients’ correspondence and prevent ‘unsuitable’ letters being sent, such as letters which highlighted the patient’s ‘mental disturbance’.^37^ Other letters kept back were those criticising the asylum.^38^ In the Cape, lunacy laws required that asylum officials had to read and comment on all patient letters, a process that was rigorously followed. However, it did not seem common practice to prevent letters from being sent.^39^

Historically, letters of complaint have been an important method for patient self-advocacy, and investigations of these complaints have sometimes led to patient victories against authority figures.^40^ Lea uses several different rhetorics in this letter to argue for his freedom. In the obvious sense, he differentiates himself from the attendants by drawing a national line between them, emphasising that he is an English soldier. Long before the war, English settlers largely held an attitude of contempt for the Boers, who were perceived as lazy, pampered by enslaved people, and doing nothing to develop the colony.^41^ British officials could not make up their minds about whether the Boers were ‘white’ or not – they represented ethnic and racial ambiguity as people who were no longer European but not African either.^42^ When social and political animosity between English settlers and Boers reached a high point during the war, it was strongly influenced by a discourse of racialism. The strand of patriotic British ‘New Journalism’ portrayed Boers as dirty, uncivilised, and animalistic.^43^ This further influenced how British settlers at the Cape viewed the Boers – as racially inferior.^44^ In his letter, when Lea makes a distinction between himself and the ‘Pro-Boer’ attendants, this is not simply a national line but a racial line. When Lea complains about the ‘persecution’ he suffers for being British, it is an outrage at the inversion of racial hierarchies expected by a British soldier serving during the war.

## Robben Island

On 12 April, Dr Dodds transferred Lea to the Robben Island Asylum, which took in severe and chronic cases of insanity.^45^ Robben Island was established in 1846 as the first and oldest lunatic asylum in the Cape, which formed part of the General Infirmary that the colonial government created to house people who suffered from leprosy, insanity, or chronic sickness.^46^ As an island 6.9 kilometres from the mainland of Cape Town, it was removed from the rest of the colony and housed patients of different races, both paying and non-paying. For most of the nineteenth century, the public viewed the island as a terrifying, brutal place where people were exiled.^47^ As a result, the asylum was unpopular among white, middle-class patients, and over the century, only patients regarded as ‘incurable cases’ were sent there.^48^ Despite this, Lea’s admittance form, signed by Robben Island’s Medical Superintendent R. Sinclair Black, states: ‘I cannot say that he is of unsound mind’.^49^

On 30 April, Lea wrote a letter to the Medical Officer of Robben Island that appears to explain this outcome. Two months after being admitted to Valkenberg, he was ‘discharged as sane and fit to travel to my wife and family in India by Dr Dodds’.^50^ He was meant to have left the colony by ship in March, but alleges that due to Dr Dodds ‘neglect of duty, I have been detained here ever since, which is illegal. Dr Dodds transferred me to a Criminal Lunatic Asylum amongst murderers and other criminals of the law, thus disgracing the British Army. Is this the treatment meted out to British soldiers after fighting for his country?’^51^

Lea shows resentment towards Dodds, who does not act in the manner Lea expects of a British doctor towards a fellow British subject in the unfamiliar terrain of the Cape. During the nineteenth century, almost all doctors at the Cape were part of a community of male, middle-class immigrants from Britain who looked at their British colleagues and institutions for inspiration and affirmation.^52^ Dodds was particularly focused on aligning the Cape’s asylum practice with Britain.^53^ Yet, Lea accuses him of illegally detaining him and relying on his Dutch and German attendants to manage his patients.

On the same day, Lea wrote another letter, this time to Prime Minister Gordon Sprigg. He writes that after trying to report him for mistreatment, Dr Dodds transferred Lea to Robben Island. He describes the ‘brutal treatment I received from his Pro-Boer attendants in the very presence of Dr Dodds. Mr MacMahon and Captain Norwood of the Royal Horse Artillery got served the same from the Boer attendants. It is a perfect disgrace should it be made known to the British Public to be half-starved and brutally ill-treated by the Boer attendants […]’.^54^

Lea accuses Dr Dodds of being complicit, perhaps even sympathising, with his attendant’s actions against his British patients, implying an unacceptable betrayal of national, and therefore racial, loyalties. He certainly thought that and subsequently wrote a letter to attorneys Van Zyl and Buisinne asking them to take his case and take action against Dr Dodds.^55^

Though he did not receive replies, the Colonial Office began to address Lea’s concerns. On the same day that he wrote to the attorneys, the Colonial Secretary wrote to the Attorney General calling for an enquiry into Lea’s complaints of mistreatment.^56^

On 23 May, Under Colonial Secretary Langham Dale wrote a letter to the Transport Officer stating that Dr Black ‘reported that the patient has practically recovered and may with safety be discharged for the purpose of proceeding to India’.[Fn fn57] ^58^ On 15 June, the Base Commandment of Robben Island notified Secretary Dale that they had organised a free passage for Lea to India at the first available opportunity.^59^ However, three days later, Base Commandment received a telegram from Robben Island stating that Lea had since ‘assaulted Dr Black’ and wrote back suggesting an enquiry into whether Lea had ‘relapsed’.^60^

The day before this telegram, Lea had written to the Colonial Secretary alleging the ‘constant drunkenness and brutality carried out’ at Robben Island. He writes, ‘I have seen by my own two eyes two patients named Scott and Putter brutally flogged by these cruel tyrants’. He names two attendants – Mr Knutt and Mr O’Dea, as having participated in this assault. He continues saying that Mr Knutt reported him on 11 June for ‘using bad language against a drunken Head Attendant’.^61^

However, a letter Lea wrote to Mr Knutt on 12 June tells a different story. Lea apologises to Knutt for having tried to assault him, asking him for forgiveness and to ‘intercede with Dr Black and ask him for mercy’s sake to not stop me from going away’.^62^ It seems clear that Lea attempted to manipulate his situation by taking on different positions depending on who he was addressing. He was desperate to be released and eager to sway people to his cause.

On 25 June, Lea wrote another letter to the Attorney General claiming that he was ‘confined in a square yard 110x60 feet, mixed along with 120 kaffirs of the criminal class. This is enough to drive any white man to further commit himself’.^63^

Lea displays the typical outrage at being confined with Black patients at this time. Indeed, this was an unusual situation. White patients were rarely confined in the same wards as Black patients, even in asylums with both patients. Physicians thought that interracial contact would cause white people to become mad through exposure to ‘primitive’ behaviour.^64^ By being confined with Black patients, it seems that Lea caused enough of a commotion that racial separation was not the priority.

Three days after Lea’s letter, George Piers wrote a letter to Secretary Noel Janisch claiming that Lea’s story was ‘absolutely untrue’. Piers claims that Lea attacked Dr Black on 11 June and was then moved to the ‘native section’, which had more secure seclusion rooms. According to Piers, Lea regretted his actions against Dr Black. He ends the letter by saying that ‘Lea is a man whose statements are not to be relied upon’.^65^

Lea’s situation appears to be getting worse at this point. At the end of June, the Base Commandant wrote again to Langham Dale, stating that the military authorities could not take responsibility for Lea during the voyage to India but that he might be able to return after the war was over if the Indian Government could arrange Lea’s care.^66^ Leaving the Cape after the war could not be the ideal solution for Lea, especially as the end was nowhere in sight. On 4 July, Dale appears to finally explain Lea’s transfer to Robben Island in the first place. Writing to the Governor, Dale claims that Lea was transferred ‘through his having threatened personal violence to Dr Dodds, in one of his fits of ungovernable rage to which he is subject […]’.^67^ Despite this, Dale continued to correspond with the Military Authorities, who insisted that Lea needed an escort for the voyage.^68^

On 10 July, Lea wrote another letter to the Colonial Secretary admitting that he had ‘used wild words towards Dr Dodds at his asylum Valkenberg’. He apologised but added that Dodds had provoked him. Lea then proceeds to describe two assaults on other patients that he witnessed. He claims that a Boer patient and a coloured patient were both assaulted by attendants:

Boer prisoner Boysens was kicked in the ribs and struck in the face by attendants Clarke and Serch and has a black eye. The other patient Andries was held down by attendants and in the presence of Dr Black and Head Attendant Knutt was allowed to be kicked by Hennigee, a half-witted nigger who is used by the attendants to punish the other unfortunates.^69^

These descriptions of abuse against patients are shocking, enough that they stirred Lea to take the sides of a Boer and a coloured patient. Though initially resentful of Boer attendants having power over him, at this point in his detainment, Lea appears to have noticed that the attendants were mistreating patients regardless of national or racial lines. Why would a British soldier try to defend a Boer prisoner? Perhaps this points to Lea’s sense of honour as a soldier. He cannot be reduced to a caricature, uncritically holding the prejudices expected of him. He aligns himself with the other ‘inmates’ as vulnerable people at risk of abuse. Although just as vulnerable, Lea takes the risk of drawing attention to these events and demanding justice, perhaps feeling that he is more capable of doing this than others.

Lea was an educated British man who knew how institutions worked. He knew the processes of filing complaints and to whom these complaints were addressed, as well as the laws surrounding his institutionalisation. Lea could advocate for himself and others in a way that was not possible for many other patients, making his case both exceptional and unsurprising. Many case studies of patients’ experiences have focused on the wealthier, more educated minority of psychiatric patients: those who could access certain privileges and leave a stronger trace in case records.^70^

These assaults deeply affected Lea, as he wrote another two letters condemning them to Premier Sprigg and Mr De Villiers, the Acting Commissioner. On 13 July, De Villiers wrote a report for an inquiry into Lea’s accusations. Still, the report is dismissive. De Villiers writes:

I am of the opinion that a certain amount of force was necessary to get Boysens to his room, but I do not think undue forced was used. With regard to the other charge of assault in the case of patient Andries, I think the attendants had to do their best to quiet down the two patients under the direction of the Head Attendant. Lea is in a very excitable condition at present, although he was rather calm when I took down his statements. He is chafing against confinement which makes him lose a good deal of self-control.^71^

Four days later, Lea wrote to De Villiers again, claiming that Dr Black encouraged his attendants to ‘aggravate’ Lea, ‘which is contrary to the Lunacy Act’.^72^ Surprisingly, Lea appears to know the details of the Lunacy Laws at the Cape. It appears that he is referencing Part IV of the *Act*, stating that:

Any officer, nurse, attendant, servant, or other person employed in any asylum or other place, or any person having the care or charge of a lunatic, or alleged lunatic, whether by reason of any contract, or any tie of relationship, or marriage, or otherwise, who shall illtreat or wilfully neglect any such lunatic or alleged lunatic shall, upon conviction, be liable to a penalty not exceeding fifty pounds, or to imprisonment, with or without hard labour, for any period not exceeding six months.^73^

However, notes surrounding these letters by De Villiers state, ‘I do not think this should be taken seriously. The patient is at present in a highly excited state of mind’.^74^

Of importance in these letters is the language used by officials to describe Lea’s emotional state, which reveals much about how these officials perceived him. In Britain at this time, an individual’s ability to control their emotions was socially important. Extreme displays of emotion were often seen as a sign of mental instability, especially in women.^75^ In Dale’s letters, Lea’s ‘fits of ungovernable rage’ are further evidence of his insanity. De Villiers describes Lea as ‘excited’ and ‘excitable’, loaded terms when describing patients. During this period, Kraepelin’s work on psychiatric classification had not taken hold in Cape asylums, and the principal diagnoses doctors made use of were ‘mania’ and ‘melancholia’.^76^ These were differential diagnoses between black and white patients, with the former almost exclusively diagnosed with mania and the latter with melancholia.

Cape psychiatrists believed that white people had more complex nervous systems and brains, and so were more vulnerable to melancholia. Black patients, in contrast, were seen as childish and less developed and more prone to simpler forms of mania.^77^ The mania diagnosis included symptoms such as restlessness, excitability, delusions, and hallucinations.^78^ In an affidavit written during the investigation at Robben Island, Dr Black writes that he initially believed Lea ‘was a man recovering rapidly from an attack of acute mania’.^79^ Here, Lea’s diagnosis relegates him to the position of a Black lunatic—disruptive, lacking control, and ultimately of an inferior mind.

Focusing on ‘excitability’ as a marker of insanity is an example of how Black patients suffered harm and injustice in Cape asylums. There are no letters by Black patients in these archives. Their life stories have been especially erased from asylum casebooks because doctors lacked the language skills to communicate with their Black patients.^80^ In diagnosing them, doctors relied on observing behaviour rather than speaking to their Black patients.^81^ This stands in contrast to the way that white patients were treated, as the casebooks reveal that doctors took more time thinking about these patients’ statements and their possible diagnoses.^82^

The primary clinical treatment used in Cape asylums at this time was ‘moral therapy’, an approach to treating mental patients that focused on compassionate care and creating a comfortable environment where patients could engage in ‘wholesome’ work and recreation.^83^ Patients who worked hard at their jobs within the asylum were understood by doctors to be on the path to recovery, unlike those patients who could not perform work due to physical or mental limitations, or those who refused to work. In Cape asylums, paying white patients had a variety of recreational pursuits in their daily program. In contrast, non-paying Black patients were employed in heavy labour for the asylum, such as farming, domestic work, and kitchen labour.^84^ Casebooks from the Robben Island Lunatic Asylum show that Black male patients were put to work in the asylum’s garden, workshops, and kitchen at much greater rates than white male patients.^85^ These patients were used as an unpaid labour force in the asylum, which reduced costs while ensuring that the asylum operated as different institutions for patients of different races and classes.^86^

Though Lea did not receive responses to his letters, the asylum administration was dealing with the matter. On 29 July, the ‘Report of Official Inspection by Mr W. Moore to Robben Island Asylum’ was sent to Dale, stating that ‘The only complaint Lea made was that he had been placed on the island and was kept there without cause’.^87^

On 6 August, Dr Black sent a detailed report titled ‘The Case of Lunatic Richard Lea’ to Noel Janisch. The report reads that Lea was transferred from Valkenberg Asylum due to ‘maniacal outbursts, and was at times most violent and threatening’. However, during his admission to Robben Island, he appeared ‘quiet, and conducted himself in a rational manner’ so that Black could not certify him insane. However, Black writes:

Over the course of a few weeks, Lea showed a change of temper, expressing delusions as regards his treatment by Dr. Dodds and using most threatening language […]. He wrote letters to lawyers in Cape Town to take legal proceedings against Dr. Dodds for detaining him. He was at the same time constantly writing letters to the Governor, the Colonial Secretary and other Officials, then began to make accusations of impropriety and conduct (sexual) against individuals on the island […]. It was reported to me, however, one morning by the Head Attendant that Lea had threatened to murder me if he got the opportunity, saying that I was in a conspiracy with Dr. Dodds to illegally detain him.

Dr Black then claims that Lea ‘attempted to make a murderous attack’ on him and made several suicide attempts. Due to this, Dr Black felt that he could not allow Lea to leave the asylum without an escort.^88^ Fortunately for Lea, a month later, a letter from Sergeant Claud Beresford Cairns arrived, claiming that he was a friend of Lea’s and would be willing to accompany him back to India.^89^ On 27 September, Acting Commissioner Jackson of Robben Island sent a letter back agreeing to the passage.^90^

On 2 October, Dr Black wrote a note that Dr Dodds and the Commissioner were attempting to locate Lea’s relatives to propose his return to India.^91^ Lea was not aware of this, as on that same day, he wrote to Chief Justice Buchanan of the Supreme Court, accusing Dr Black of treating him ‘as a dangerous lunatic’. He continues:

There are murderers and patients placed at the same table as we honourable men. It is a perfect disgrace to witness such gross irregularities from Dr. Black and the attendants. How could honourable men face the world again and have it said to be an associate with the criminal insane of South Africa? I humbly beg of your Lordship to bring my case before Parliament and have these inhuman fiends brought to public justice.^92^

At the heart of Lea’s desperation is his refusal to associate himself with patients he believes are not ‘honourable men’. Lea holds himself to a code of conduct; he is a soldier, a man who clearly has a strong sense of justice. He defends his fellow patients, even if they are his ‘enemies’. He acts out against those he believes are wrongdoers, even as it confirms their opinions about his mental state.

## Truth, lies, and the media

On 4 October 1901, *The Owl*, a newspaper in the Cape Colony, published a letter detailing Lea’s allegations of abuse.^93^ The letter reads that while at Valkenberg, Lea witnessed a ‘cruel and cowardly assault made by attendants on Sergeant Stanley, of the Army Service Corps […]. Sergeant Lea took his comrade’s part, and was likewise assaulted, and then transferred to Robben Island Asylum by Dr. Dodds’.

In the Valkenberg casebook, Dr Dodds describes Stanley as an English soldier suffering from ‘General Paralysis of the Insane’. Dodds writes that Stanley is ‘very troublesome, continually interfering with other patients and getting into rows. Consequently, he’s frequently gotten marks of injury on his face, which are his own fault entirely’.^94^ Attached to these case notes is a document with an affidavit from Charles L, one of the Valkenberg attendants. He writes that ‘Lea’s statement is untrue […]. I never saw any one of the attendants ill-treat Stanley. He was generally fighting with one or other of the patients, at one time he had a black eye given to him by one of the patients’.^95^

Of course, there is no way of verifying any of these claims. Whether Stanley was assaulted or not, this case shows that Lea was quick to point out perceived wrongs committed against patients from the beginning of his confinement. Three attendants appear to have signed *The Owl* letter; however, notes in the casebook show that one attendant declared that he did not sign it and knew nothing about Sergeant Lea.

On 15 October, attorneys Van Zyl and Buisinne sent a letter to Noel Janisch stating that Lea was contemplating an application to the Supreme Court regarding his detainment in the asylum.^96^ The very next day, Lea presented his petition to the Supreme Court. *The Cape Times* reported on this petition, publishing an article stating that asylum officials were planning to move Lea to a new location ‘beyond the jurisdiction of the Court’.^97^ The Supreme Court then ruled that the Superintendents of Robben Island Asylum had to show cause for the removal of Lea from the jurisdiction of the Court.^98^

That same day, Langham Dale sent a letter to the Commissioner informing him that Lea’s passage to India was booked for the next day at 11:30 am.^99^ However, once Dale read about the Supreme Court petition, he telegraphed the Commissioner to cancel the passage.^100^ On 23 October, Acting Commissioner Jackson wrote a report about the Lea case, stating:

I have several times interviewed Lea, on one occasion in the presence of Dr Murray, one of the recognised Official Visitors. I can testify from personal observation that Lea develops an excited mental condition, taking the shape of suspicion of all the attendants who he stated have on one or more occasions treated the patients under their charge in a cruel manner. I am satisfied that there is no ground for the accusations and that Lea is labouring under delusions.^101^

On 25 October, the case notes show an affidavit by three attendants stating that they had nothing to do with the letter they supposedly signed in *The Owl.* ^102^ They suspected that Lea forged the letter himself. By this point, Lea seems to have discovered that Cape officials were trying to organise his departure to India, as on 31 October, he sent a telegram to Noel Janisch saying, ‘Sir, I have stopped court case. Kindly send me away’.^103^ On 11 November, Janisch wrote to Lea’s wife about removing him to India, warning her:

From the Medical Reports it would appear that he is still subject to violent outbreaks of a maniacal nature and has not yet entirely recovered his sanity. I shall be glad to learn whether, if arrangements were made with the Military Authorities for Mr. Lea’s passage to India, you would be prepared to take charge of him.^104^

On 19 November, Janisch wrote to the Commissioner giving authorisation for Lea’s discharge from the asylum under the ‘General Regulation No. 2’ of the *Lunacy Act of 1897*, which allowed for patients to be discharged as ‘unrecovered’*.* ^105^ The regulation states that:

When a lunatic who is detained in an asylum or elsewhere […] is in such a mental condition that, in the opinion of the Asylum Medical Officer or other Medical Attendant he no longer requires special treatment, and may be safely handed over to the care of relatives, or other approved persons, application for his discharge may be made to the Colonial Secretary, who may thereupon authorise such discharge accordingly.^106^

Finally, on 12 December, Langham Dale wrote to Lea’s wife that Lea and his escort had sailed from Durban for Bombay on the *S.S. Columbian* on 1 December 1901.^107^ No more was heard from Richard Lea.

## Conclusion

The case of Richard Lea shows a long-winded, sometimes dramatic, often tedious attempt by an asylum patient to secure his release and return home. It is clear that Lea caused a lot of trouble for colonial officials at the Cape, and it might be argued that his release depended more on a desire to be rid of a troublemaker, rather than a genuine belief that he would be better off at home. This story would undoubtedly be very different if Lea were not who he was.

As an English soldier who knew the rules of a bureaucracy and the codes for filing complaints, he was unusually equipped to advocate for his liberation. His reasons for defending fellow patients are complex; these might have been empathetic attempts at standing up for the vulnerable or opportunities for stirring up more cases of abuse to complain about. Regardless, Lea knew how to work the system he was in. His context was an environment at war, a rapidly changing colony soon to be united into one country, a lunatic asylum, and a bureaucratic legal system, among others. He lived in a changing, turbulent, unpredictable world, which shows in his letters.

The histories present within colonial asylums present challenges to the historian. The traces of patients’ voices within the archive contain truths and fictions, ethical dilemmas, and discursive structures that shape the way historians write about them.^108^ The contradictions and tensions in this narrative resist the possibility of reading this simplistically. Reading these various letters produces a narrative full of contradictions and ethical tensions, a story that rejects a simplistic reading of the sources. It is never evident if anyone is telling the truth, and the truth does not matter.

There was never a ‘great confinement’ in South African asylums, where the mentally ill were systematically detained through the incredible power of the state.^109^ Still, there are few sources to show what people experienced in these institutions. The case of Richard Lea is an exceptional story that shows how one patient navigated a system that often seems too opaque and chaotic for the historian to understand. It gives insight into the inner workings of the asylum and the legal policies of a colony at war, showcasing the changes taking place in a turbulent South Africa. One is given a view into the personal lives of patients and attendants, the medical rules doctors followed, and instances of racism, unexpected solidarity, and loneliness. A rare example of the patient’s voice, in this casebook, Richard Lea is a ghost who never stops calling out.

## Footnotes

1

Iain R. Smith , ‘The Origins of the South African War (1899–1902): A Re-Appraisal’, South African Historical Journal, 22, 1 (1990), 25 .

2 See Louis Venter and André Wessels , ‘British Soldiers’ Anglo-Boer War Experiences as Recorded in Their Diaries’, South African Journal of Cultural History, 36, 2 (2022), 63–83 ; Helen M. Ross, ‘A Woman’s World at a Time of War: An Analysis of Selected Women’s Diaries during the Anglo-Boer War 1899–1902’ (unpublished PhD thesis: Stellenbosch University, 2006); and Elizabeth van Heyningen , ‘The Voices of Women in the South African War’, South African Historical Journal, 41, 1 (1999), 22–43 10.1080/0258247990867188222432170.

3 In this paper, Richard Lea’s name is written in full. This may be controversial in the ethics practices within the history of psychiatry. Some scholars argue that using pseudonyms is dehumanising, while others advocate for their use for the sake of privacy. In South Africa, there is no defined period of restriction on medical records. Richard Lea was not shy or hesitant about publicly referencing his institutionalisation. Due to this, it feels correct to use his name.

4

Katherine Rawling , ‘Patient photographs, patient voices: Recovering patient experience in the nineteenth-century asylum’, in R. Ellis , S. Kendal , and S.J. Taylor (eds.), Voices in the History of Madness: Personal and Professional Perspectives on Mental Health and Illness (Cham: Springer Nature Switzerland, 2021), 237 .

5
*Ibid.*, 318.

6

Alexandra Bacopoulos-Viau and Aude Fauvel , ‘The Patient’s Turn: Roy Porter and Psychiatry’s Tales, Thirty Years on’, Medical History, 60, 1 (2016), 1 10.1017/mdh.2015.65PMC484738126651185.

7

Louise Wannell , ‘Patients’ Relatives and Psychiatric Doctors: Letter Writing in the York Retreat, 1875–1910’, Social History of Medicine,20, 2 (2007), 300 10.1093/shm/hkm04318605330.

8

Catharine Coleborne , Madness in the Family: Insanity and Institutions in the Australasian Colonial World, 1860–1914 (London: Palgrave Macmillan, 2009), 91 .

9

Effie Karageorgos , ‘“The Unseen Enemy Persists”: Delusion, Trauma and the South African War in Australian Asylum Case Notes’, Social History Of Medicine,36, 2 (2023), 319 10.1093/shm/hkac049PMC1039236037533511 and Rory du Plessis , ‘Tells his Story Quite Rationally and Collectedly: Examining the Casebooks of the Grahamstown Lunatic Asylum, 1890–1910, for Cases of Delusion Where Patients Voiced their Life Stories’, in R. Ellis , S. Kendal , and S.J. Taylor (eds.), Voices in the History of Madness: Personal and Professional Perspectives on Mental Health and Illness (Cham: Springer Nature Switzerland, 2021), 139 .

10

Anne Hanley and Jessica Meyer (eds), Patient Voices in Britain, 1840–1948 (Manchester: Manchester University Press, 2021), 2.

11 Karageorgos, *op. cit.* (note 9), 321.

12 Coleborne, *op. cit.* (note 8), 99.

13
*Ibid.*, 9.

14

Roy Porter , ‘The Patient’s View: Doing Medical History from Below’, Theory and Society, 14, 2 (1985), 181 11618212.

15

Sally Swartz , ‘Lost Lives: Gender, History and Mental Illness in the Cape, 1891–1910’, Feminism & Psychology, 9, 2 (1999), 152 .

16
*Ibid.*, 153.

17

Rory du Plessis , ‘Photographs from the Grahamstown Lunatic Asylum, South Africa, 1890–1907’, Social Dynamics,40, 1 (2014), 13 .

18

Julie Parle , States of Mind: Searching for Mental Health in Natal and Zululand, 1868–1918 (Scottsville: University of KwaZulu-Natal Press, 2007), 6.

19 Rory du Plessis, *op. cit.* (note 9), 139.

20 Rory du Plessis, Pathways of Patients at the Grahamstown Lunatic Asylum, 1890 to 1907 (Pretoria: Pretoria University Law Press, 2020), 6.

21 The casebooks used as sources for this paper form part of the Valkenberg Asylum collection at the Western Cape Archives and Records Services. As Valkenberg was a white-only institution at this time, available letters were written by white patients.

22
*The Owl*, 4 October 1901, CO 7501, Western Cape Archives and Records Service, Cape Town, South Africa.

23

J.C. de Villiers , ‘Hospitals in Cape Town during the Anglo-Boer War,’ South African Medical Journal,89, 1 (1999), 81 10070419.

24
*Ibid.*, 86.

25
*The Cape Times*, 3 October 1901, CO 7501, Western Cape Archives and Records Service, Cape Town, South Africa.

26 Medical Certificate (Lunacy Form No. 1), 13 January 1901, CSC 2/6/1/208, Western Cape Archives and Records Service, Cape Town, South Africa.

27 ‘The first wounded to arrive at the Cape: a ward in Wynberg Camp’, drawn by Percy F. S. Spence from a sketch by H. Egersdorfer. 1900. *Wellcome Collection.*

28

Richard C. Keller , ‘Taking Science to the Colonies: Psychiatric Innovation in France and North Africa’, in Sloan Mahone and Megan Vaughan (eds.), Psychiatry and Empire (New York: Palgrave Macmillan, 2007), 23 .

29

Sloan Mahone , ‘East African Psychiatry and the Practical Problems of Empire,’ in Sloan Mahone and Megan Vaughan (eds.), Psychiatry and Empire (New York: Palgrave Macmillan, 2007), 48 .

30 Medical Statement (Lunacy Form no. 7), 13 January 1901, CSC 2/6/1/208, Western Cape Archives and Records Service, Cape Town, South Africa.

31 Karageorgos, *op. cit.* (note 9), 333.

32

Rory Du Plessis , ‘A Hermeneutic Analysis of Delusion Content from the Casebooks of the Grahamstown Lunatic Asylum, 1890–1907,’ South African Journal of Psychiatry, 25 (2019), 3 10.4102/sajpsychiatry.v25i0.1263PMC642453630899582.

33 Medical Statement (Lunacy Form no. 7), 13 January 1901, CSC 2/6/1/208, Western Cape Archives and Records Service, Cape Town, South Africa.

34

Sally Swartz , ‘Multiple Voices and Plausible Claims: Historiography and Colonial Lunatic Asylum Archives’, Medicine and Colonialism, 148 (2015).

35 R. Lea, Letter, 25 February 1901, CO 7501, Western Cape Archives and Records Service, Cape Town, South Africa.

36 R. Lea, Letter, 9 March 1901, CO 7501, Western Cape Archives and Records Service, Cape Town, South Africa.

37

Allan Beveridge , ‘Life in the Asylum: Patients’ Letters from Morningside, 1873–1908’, History of Psychiatry,9, 36 (1998), 434 10.1177/0957154X980090360211623612.

38
*Ibid.*, 435.

39

Sally Swartz , ‘Multiple voices and plausible claims: historiography and colonial lunatic asylum archives’, in Poonam Bala (ed.), Medicine and Colonialism (London: Routledge, 2015), 152 .

40 Hanley and Meyer, *op. cit.* (note 10), 9.

41

Robert Ross , Status and Respectability in the Cape Colony, 1750–1870: A Tragedy of Manners (Cambridge: Cambridge University Press, 2004), 42 .

42

Melvin van Wyk Smith , ‘The Boers and the Anglo-Boer War (1899–1902) in the twentieth-century moral imaginary’, Victorian Literature and Culture,31, 2 (2003), 433 .

43 Karageorgos, *op. cit.* (note 9), 329.

44 van Wyk Smith, *op. cit.* (note 42), 440.

45 E. Jackson, Report, 23 October 1901, CO 7501, Western Cape Archives and Records Service, Cape Town, South Africa.

46

Harriet Deacon , ‘Madness, Race and Moral Treatment: Robben Island Lunatic Asylum, Cape Colony, 1846–1890’, History of Psychiatry, 7, 26 (1996), 287 11613412 10.1177/0957154X9600702606.

47
*Ibid.*, 288.

48
*Ibid.*, 293.

49 Lunacy Form No. 38, 22 April 1901, Western Cape Archives and Records Service, Cape Town, South Africa.

50 R. Lea, Letter, 30 April 1901, CO 7501, Western Cape Archives and Records Service, Cape Town, South Africa.

51 R. Lea, Letter, 30 April 1901, CO 7501, Western Cape Archives and Records Service, Cape Town, South Africa.

52 Harriet Deacon, ‘Racism and Medical Science in South Africa’s Cape Colony’, *Osiris,* 15 (2000),196.

53

Sally Swartz , ‘Changing Diagnoses in Valkenberg Asylum, Cape Colony, 1891–1920: A Longitudinal View’, History of Psychiatry, 6 (1995), 433 10.1177/0957154X950060240211609002.

54 R. Lea, Letter, 30 April 1901, CO 7501, Western Cape Archives and Records Service, Cape Town, South Africa.

55 R. Lea, Letter, 4 May 1901, CO 7501, Western Cape Archives and Records Service, Cape Town, South Africa.

56 Colonial Secretary, Letter, 30 April 1901, CO 7501, Western Cape Archives and Records Service, Cape Town, South Africa.

57

Rand McNally and Company , Maps of Africa to 1900 (Urbana, IL: Rare Book & Manuscript Library, University of Illinois at Urbana-Champaign Library, 1887).

58 Langham Dale, Letter, 23 May 1901, CO 7501, Western Cape Archives and Records Service, Cape Town, South Africa.

59 Base Commander, Letter, 15 June 1901, CO 7501, Western Cape Archives and Records Service, Cape Town, South Africa.

60 H. Tucker, Letter, 19 June 1901, CO 7501, Western Cape Archives and Records Service, Cape Town, South Africa.

61 R. Lea, Letter, 18 June 1901, CO 7501, Western Cape Archives and Records Service, Cape Town, South Africa.

62 R. Lea, Letter, 12 June 1901, CO 7501, Western Cape Archives and Records Service, Cape Town, South Africa.

63 R. Lea, Letter, 25 June 1901, CO 7501, Western Cape Archives and Records Service, Cape Town, South Africa.

64 Swartz, *op. cit.* (note 39), 148.

65 G. Piers, Letter, 28 June 1901, CO 7501, Western Cape Archives and Records Service, Cape Town, South Africa.

66 Base Commandant, Letter, 29 June 1901, CO 7501, Western Cape Archives and Records Service, Cape Town, South Africa.

67 L. Dale, Letter, 4 July 1901, CO 7501, Western Cape Archives and Records Service, Cape Town, South Africa.

68 L. Dale, Letter, 5 July 1901, CO 7501, Western Cape Archives and Records Service, Cape Town, South Africa.

69 R. Lea, Letter, 10 July 1901, CO 7501, Western Cape Archives and Records Service, Cape Town, South Africa.

70 Hanley and Meyer, *op. cit.* (note 10), 2.

71 De Villiers, Letter, 13 July 1901, CO 7501, Western Cape Archives and Records Service, Cape Town, South Africa.

72 R. Lea, Letter, 17 July 1901, CO 7501, Western Cape Archives and Records Service, Cape Town, South Africa.

73
*The Lunacy Act 1897*, CO 7597, Western Cape Archives and Records Service, Cape Town, South Africa.

74 De Villiers, notes, 15 July 1901, CO 7501, Western Cape Archives and Records Service, Cape Town, South Africa.

75 Coleborne, *op. cit.* (note 8), 99.

76 Swartz, *op. cit.* (note 53), 439.

77
*Ibid.*, 441.

78
*Ibid.*, 436.

79 R. Sinclair Black, Affidavit, 22 October 1901, CSC 2/6/1/208,Western Cape Archives and Records Service, Cape Town, South Africa.

80 Rory du Plessis, *op. cit.* (note 9), 139.

81 Swartz, *op. cit.* (note 39), 149.

82 du Plessis, *op. cit.* (note 9), 142.

83 Karageorgos, *op. cit.* (note 9), 328.

84

Rory du Plessis , ‘The Principles and Priorities of Dr TD Greenlees, Medical Superintendent of the Grahamstown Lunatic Asylum, 1890–1907’, Historia, 60, 1 (2015), 35 .

85 Head Attendant’s Report, 1899, MSB 397, National Library of South Africa, Cape Town, South Africa.

86 Rory du Plessis, ‘The principles and priorities of Dr T.D. Greenlees’, *Historia,* 60, 1 (2015), 41.

87 W. Moore, ‘The Report of Official Inspection by Mr. W. Moore to Robben Island Asylum’, 29 July 1901, CO 7501, Western Cape Archives and Records Service, Cape Town, South Africa.

88 S. Black, ‘The Case of Lunatic Richard Lea’, 6 August 1901, CO 7501, Western Cape Archives and Records Service, Cape Town, South Africa.

89 C.B. Cairns, Letter, 19 September 1901, CO 7501, Western Cape Archives and Records Service, Cape Town, South Africa.

90 Jackson, Letter, 27 September 1901, CO 7501, Western Cape Archives and Records Service, Cape Town, South Africa.

91 S. Black, Letter, 2 October 1901, CO 7501, Western Cape Archives and Records Service, Cape Town, South Africa.

92 R. Lea, Letter, 2 October 1901, CO 7501, Western Cape Archives and Records Service, Cape Town, South Africa.

93
*The Owl*, 4 October 1901, CO 7501, Western Cape Archives and Records Service, Cape Town, South Africa.

94 W.J. Dodds, Casebook Notes, 15 February 1900, CO 7501, Western Cape Archives and Records Service, Cape Town, South Africa.

95 Charles L., Affidavit, 1901, CO 7501, Western Cape Archives and Records Service, Cape Town, South Africa.

96 Van Zyl and Buisinne, Letter, 15 October 1901, CO 7501, Western Cape Archives and Records Service, Cape Town, South Africa.

97
*The Cape Times*, 3 October 1901, CO 7501, Western Cape Archives and Records Service, Cape Town, South Africa.

98 C.F.K Murray, Letter, 17 October, CO 7501, Western Cape Archives and Records Service, Cape Town, South Africa.

99 Langham Dale, Letter, 16 October 1901, CO 7501, Western Cape Archives and Records Service, Cape Town, South Africa.

100 Langham Dale, Letter, 17 October 1901, CO 7501, Western Cape Archives and Records Service, Cape Town, South Africa.

101 E. Jackson, Report, 23 October 1901, CO 7501, Western Cape Archives and Records Service, Cape Town, South Africa.

102 Affidavit, 25 October 1901, CO 7501, Western Cape Archives and Records Service, Cape Town, South Africa.

103 R. Lea, Telegram, 31 October 1901, CO 7502, Western Cape Archives and Records Service, Cape Town, South Africa.

104 Noel Janisch, 11 November 1901, CO 7502, Western Cape Archives and Records Service, Cape Town, South Africa.

105 Noel Janisch, 19 November 1901, CO 7502, Western Cape Archives and Records Service, Cape Town, South Africa.

106
*The Lunacy Act 1897*, CO 7597, Western Cape Archives and Records Service, Cape Town, South Africa.

107 L. Dale, Letter, 12 December 1901, CO 7502, Western Cape Archives and Records Service, Cape Town, South Africa.

108 Swartz, *op. cit.* (note 39), 143.

109

Megan Vaughan , Curing Their Ills: Colonial Power and African Illness (Stanford: Stanford University Press, 1991), 101 .

